# The Cholesterol Biosynthesis Pathway Plays an Important Role in Chemotherapeutic Drug Response and Metastasis in High-Grade Osteosarcoma

**DOI:** 10.3390/cells14130993

**Published:** 2025-06-29

**Authors:** Amonnat Sukhamwang, Dumnoensun Pruksakorn, Pornngarm Dejkriengkraikul, Apiwat Sangphukieo, Sivamoke Dissook, Supachai Yodkeeree

**Affiliations:** 1 Department of Biochemistry, Faculty of Medicine, Chiang Mai University, Chiang Mai 50200, Thailand; amonnat_s@cmu.ac.th (A.S.); pornngarm.d@cmu.ac.th (P.D.); sivamoke.dis@cmu.ac.th (S.D.); 2 Center of Multidisciplinary Technology for Advanced Medicine (CMUTEAM), Faculty of Medicine, Chiang Mai University, Chiang Mai 50200, Thailand; dumnoensun.p@cmu.ac.th (D.P.); apiwat.sang@cmu.ac.th (A.S.); 3 Musculoskeletal Science and Translational Research (MSTR) Center, Faculty of Medicine, Chiang Mai University, Chiang Mai 50200, Thailand; 4 Department of Orthopedics, Faculty of Medicine, Chiang Mai University, Chiang Mai 50200, Thailand; 5 Anticarcinogenesis and Apoptosis Research Cluster, Faculty of Medicine, Chiang Mai University, Chiang Mai 50200, Thailand

**Keywords:** osteosarcoma, transcriptomics, RNA-seq, cholesterol biosynthesis, drug resistance

## Abstract

High-grade osteosarcoma (HGOS) is the most common primary malignant bone tumor in children and adolescents. Poor response to chemotherapy is linked to worse prognosis and increased risk of recurrence and metastasis. However, current assessment methods, such as tumor necrosis evaluation, are time-consuming and delay treatment decisions. Thus, identifying molecular pathways and predictive biomarkers is essential for guiding early therapeutic strategies. In this study, RNA-seq analysis of HGOS tissues revealed enrichment of cholesterol biosynthesis and mitotic pathways in poor responders. Additionally, high *HMGCR* expression, as analyzed from TCGA data, was associated with poor prognosis in sarcoma. Functional validation using SaOS-2 cells, which exhibited poor drug sensitivity and elevated *HMGCR* levels, demonstrated that simvastatin enhanced the efficacy of cisplatin and doxorubicin by inducing mitochondrial-mediated apoptosis and downregulating anti-apoptotic proteins. Simvastatin also reduced cell migration and invasion by suppressing epithelial–mesenchymal transition and extracellular matrix degradation. Mechanistically, simvastatin disrupted Ras prenylation and inhibited downstream oncogenic signaling pathways, including Akt/mTOR and Akt/GSK3, which regulate survival and metastasis-associated gene expression. These findings suggest that the cholesterol biosynthesis pathway particularly plays a critical role in chemoresistance and metastasis in HGOS and may serve as a promising predictive molecular target for guiding early therapeutic strategies.

## 1. Introduction

Originating from primitive mesenchymal cells, osteosarcoma (OS) represents the most common primary malignant bone tumor in children and adolescents [1]. Patients diagnosed with high-grade osteosarcoma (HGOS) undergo neoadjuvant multi-agent chemotherapy, which consists of methotrexate, doxorubicin, and cisplatin (MAP), followed by surgical resection [2]. However, the efficacy of this treatment varies considerably among patients, and a subset exhibits intrinsic resistance. While the 5-year survival rate for localized OS is approximately 75% [3], it significantly decreases to 15–30% in patients presenting with metastatic disease [4]. Chemotherapy resistance remains a critical obstacle to achieving optimal treatment outcomes in HGOS.

Chemotherapy drug response in HGOS varies significantly among individuals due to a range of molecular mechanisms, including genetic mutations, epigenetic modifications, altered drug transport, enhanced DNA repair, dysregulated apoptosis, and changes in cell signaling pathways [5]. Overexpression of drug efflux transporters such as P-glycoprotein, MRP1, and BCRP leads to decreased intracellular drug accumulation, contributing to multidrug resistance [6]. Enhanced DNA repair mechanisms, particularly involving ERCC1, ERCC2, and BRCA1/2, can repair chemotherapy-induced DNA damage, reducing drug efficacy [5]. Activation of survival signaling pathways, including PI3K/AKT/mTOR and PI3K/AKT/GSK3 pathways, promotes cancer cell proliferation and survival from chemotherapeutic drugs [7]. Apoptosis evasion, through overexpression of anti-apoptotic proteins or suppression of apoptotic pathways, further contributes to resistance. Other contributing factors include metabolic reprogramming, epithelial–mesenchymal transition (EMT), and the presence of cancer stem cells, all of which enhance the ability of tumors to resist chemotherapy and promote relapse [8]. Therefore, identifying molecular factors that can predict the efficacy of neoadjuvant chemotherapy is urgently needed to improve prognosis and guide personalized treatment strategies.

Assessing the response to chemotherapy plays a crucial role in both the prognosis and clinical management of OS patients. The most widely used predictive marker for localized tumor response to chemotherapy is the percentage of tumor necrosis. A pathological assessment indicating >90% tumor necrosis defines a good responder, whereas <90% tumor necrosis characterizes a poor responder. Patients with a poor response to chemotherapy are generally associated with poorer prognosis and an increased risk of disease recurrence and metastasis. However, the assessment of tumor cell necrosis has certain limitations, including the extended evaluation time, which delays outcome determination and is not conducive to subsequent treatments [9]. Therefore, identifying molecular pathways and genes associated with chemotherapy response is essential for developing predictive biomarkers that can guide treatment decisions before chemotherapy initiation. To our knowledge, there are no effective diagnostic markers for predicting MAP response in HGOS cancer patients. In this study, RNA sequencing (RNA-Seq) was performed on pre-chemotherapy OS patient samples to compare the transcriptomic profiles between good responders and poor responders, with the aim of identifying potential molecular markers associated with treatment outcomes. Furthermore, we investigated the molecular mechanisms and biological pathways associated with chemotherapy response using osteosarcoma cell lines.

## 2. Materials and Methods

### 2.1. Reagents and Chemicals

Dulbecco’s Modified Eagle’s Medium (DMEM), penicillin–streptomycin solution, and trypsin-EDTA were obtained from Gibco (Grand Island, NY, USA). Fetal bovine serum (FBS) was sourced from Hyclone (Logan, UT, USA). MitoView™ 633 dye was purchased from Biotium (Fremont, CA, USA). The FITC Annexin V apoptosis detection kit was acquired from Elabscience Biotechnology (Houston, TX, USA). Antibodies targeting N-cadherin, c-FLIP, c-IAP2, BCL-2, BCL-XL, phospho-GSK3, total GSK3, Akt, and β-actin were obtained from Cell Signaling Technology (Danvers, MA, USA), while antibodies against fibronectin, claudin-1, uPA, MT1-MMP, cleaved caspase-3, phospho-mTOR, total mTOR, and phospho-Akt were provided by Abclonal (Woburn, MA, USA). Antibodies for PARP-1, uPAR, and KRas detection were purchased from Santa Cruz Biotechnology (Santa Cruz, CA, USA). Enhanced chemiluminescence (ECL) reagent was supplied by Bio-Helix (New Taipei, Taiwan). Matrigel matrix was obtained from Becton Dickinson (Bedford, MA, USA). Cisplatin was sourced from Sigma-Aldrich (St. Louis, MO, USA), and doxorubicin hydrochloride was purchased from LC Laboratories (Woburn, MA, USA).

### 2.2. Patients

Biopsy specimens from nine patients with high-grade osteosarcoma (stage IIB–III) were collected under pre-chemotherapy conditions before the initiation of neoadjuvant treatment. Diagnoses were confirmed between 2015 and 2019 at Maharaj Nakorn Chiang Mai Hospital, Thailand. Written informed consent was secured from all study participants. For individuals under 18 years of age, consent was duly obtained from their parents or legal guardians. Tissue samples were collected pursuant to formal approval by the Research Ethics Committee, Faculty of Medicine, Chiang Mai University (Approval No. ORT-2563-07122). Tissue specimens were immediately cryopreserved at −80 °C within 30 min of surgical excision and preserved for subsequent use. Tissue samples were categorized into two groups based on the percentage of tumor necrosis following treatment. Samples with ≥90% tumor necrosis were classified as good responders, while those with ≤60% tumor necrosis were classified as poor responders. To determine tumor necrosis percentages, tissue samples were collected post-chemotherapy completion.

### 2.3. Cell Cultures

Osteosarcoma cell lines, including 143B (CRL-8303) and SaOS-2 (HTB-85), were purchased from ATCC (Manassas, VA, USA), while U2OS (CLS-300364) was obtained from Cell Lines Service (GmbH, Eppelheim, Germany). All cell lines were maintained in Dulbecco’s Modified Eagle Medium (DMEM) supplemented with 10% fetal bovine serum (FBS) and 1% penicillin–streptomycin, under standard culture conditions at 37 °C in a humidified incubator with 5% CO_2_ [10].

### 2.4. Chemosensitivity Testing by MTT Colorimetric Assay

Drug sensitivity was determined using a 3-(4,5-dimethylthiazol-2-yl)-2,5-diphenyltetrazolium bromide (MTT) assay. SaOS-2, U2OS, and 143B cells were seeded in 96 well-plates at a density of 3 × 10^3^, 2.5 × 10^3^, and 7.5 × 10^2^ cells/well, respectively, in a culture medium. The cells were incubated at 37 °C, 5% CO_2_ for 72 h with cisplatin (0, 0.1, 0.5, 1, 10, and 25 μM) or doxorubicin (0, 1, 10, 100, 500, and 1000 nM). Following treatment, 10 μL of MTT solution (0.5 mg/mL in PBS) was added to each well, and the cells were incubated for an additional 4 h to allow for formazan crystal formation. After incubation, the culture medium was carefully removed, and 200 μL of DMSO was added to each well to solubilize the formazan crystals. The absorbance was measured at 540 nm with a reference wavelength of 630 nm using a UV-visible spectrophotometer. Each condition was tested in triplicate. Cell viability was calculated by comparing absorbance values to those of untreated controls and expressed as a percentage of control viability. To assess the effects of combination treatments, SaOS-2 and 143B cells were co-treated with varying concentrations of cisplatin or doxorubicin with or without simvastatin (0–3 µM). Cells were incubated at 37 °C in 5% CO_2_ for 72 h. The cell visibility was determined by MTT assay as described above.

### 2.5. Total RNA Isolation and Expression Analysis

The patient biopsy tissues were extracted using NucleoZOL reagent (Macherey-Nagel™, Düren, Germany), following the manufacturer’s protocol. mRNA sequencing libraries were then constructed, and the transcriptome was performed using an Illumina sequencing platform (Macrogen Inc., HiSeq 2500, Seoul, Republic of Korea).

### 2.6. RNA Sequencing Data Analysis

Raw mRNA sequencing data from MACROGEN Co., Ltd. (Seoul, Republic of Korea) were processed using an advanced bioinformatics workflow to ensure robust transcript analysis. The pipeline began with quality control using Cutadapt (v3.4) to trim adapter sequences and remove low-quality bases. To ensure precise mapping, cleaned reads were aligned to the human reference genome (hg38) using HISAT2 (v2.2.1). SAMtools (v1.12) was then used to sort and convert SAM files into BAM format, preparing the data for downstream analysis. Gene expression quantification and transcript assembly were performed using StringTie (v2.1.4), which generated gene transfer format (GTF) files for each sample. The resulting GTF files from each sample were merged to create a comprehensive transcriptome assembly, which was then compared against the reference annotation using GffCompare (v0.11.6). During this integration process, the default MSTRG identifiers assigned by StringTie were converted into gene names using the IsoformSwitchAnalyzeR R package (v2.6.0). Differential expression analysis was performed in the R environment using DESeq2 (v1.34.0). Genes with a |log2FC| ≥ 1.25 and a *p*-value < 0.05 were classified as differentially expressed genes. The volcano and PCA plot were created using the EnhancedVolcano R package (v1.10.0) and factoextra (v1.0.7), respectively.

### 2.7. Gene Ontology and Pathway Enrichment Analysis

The enrichment analyses including Gene Ontology, and Reactome pathways were conducted via the DAVID knowledge base (v2025_1) and visualized using the ggplot2 R package (v3.5.2).

### 2.8. Protein–Protein Interaction and Identification of Hub Genes and Subnetwork Analysis

Protein–protein interaction (PPI) networks were generated using Cytoscape software (version 3.10.3). The Molecular Complex Detection (MCODE, version 2.0.3) plugin was applied to identify densely connected gene clusters within the network. Subsequently, pathway enrichment analysis was performed using the ClueGO plugin (version 2.5.10) in Cytoscape, which visualized and grouped functionally related Gene Ontology (GO) terms into biologically meaningful networks.

### 2.9. Determination of Gene Expressions by RT-qPCR Analysis

Total RNA was extracted from osteosarcoma cell lines using NucleoZOL reagent (Macherey-Nagel™, Düren, Germany), following the manufacturer’s instructions. RNA was also extracted from patient biopsy tissues for comparative analysis. The concentration and purity of total RNA were assessed using a NanoDrop™ 2000/2000c spectrophotometer (Thermo Fisher Scientific, Waltham, MA, USA). Reverse transcription was performed using the ReverTra Ace™ qPCR RT Master Mix with gDNA Remover (TOYOBO, Osaka, Japan) to synthesize complementary DNA (cDNA). Quantitative real-time PCR (qRT-PCR) was conducted using the THUNDERBIRD™ Next SYBR^®^ qPCR Mix (TOYOBO, Osaka, Japan) on an Applied Biosystems QuantStudio 6 Flex system (software v.1.0, Waltham, MA, USA). Gene expression levels were calculated using the 2^−ΔΔCT^ method, with GAPDH serving as the internal normalization control.

### 2.10. The Correlation of HMGCR Expression with Overall Survival and Disease-Specific Survival in Sarcoma Samples

Survival analysis for *HMGCR* mRNA expression was performed using publicly available datasets from cBioPortal for Cancer Genomics. Data from the TCGA PanCancer Atlas Sarcoma cohort was utilized. The correlation between *HMGCR* expression and both overall survival (OS) and disease-specific survival (DSS) was analyzed using the cBioPortal platform. Kaplan–Meier plots were generated, and statistical significance was assessed to evaluate the prognostic impact of *HMGCR* expression in sarcoma tissue samples.

### 2.11. Apoptosis Assay

Following the manufacturer’s instructions, the Annexin V-FITC/PI Apoptosis Detection Kit FITC (Elabscience Biotechnology Inc., Houston, TX, USA) was used to examine early/late apoptotic and necrotic cells. SaOS-2 cells were treated with cisplatin 5 μM or doxorubicin 150 nM with or without simvastatin 3 μM for 48 h, and then collected with trypsin and rinsed with PBS. Following incubation, cells were stained with 2.5 µL of propidium iodide (PI) and 2.5 µL of Annexin V-FITC for 15 min at room temperature. The stained cells were subsequently analyzed by flow cytometry, and the data were processed using CytExpert for DxFLEX software (v2.0.2.18).

### 2.12. Mitochondrial Membrane Potential

Mitochondrial membrane potential was evaluated using MitoView™ 633 (Biotium, Fremont, CA, USA), following the manufacturer’s instructions. Briefly, SaOS-2 cells were treated with 5 μM cisplatin or 150 nM doxorubicin, with or without 3 μM simvastatin, for 48 h. After treatment, cells were harvested by trypsinization and incubated with 100 nM MitoView™ 633 in serum-free DMEM for 20 min at 37 °C in the dark under 5% CO_2_. Subsequently, cells were washed with PBS, and fluorescence was measured using flow cytometry at 638/660 nm (excitation/emission). Data analysis was performed with CytExpert for DxFLEX software (v2.0.2.18).

### 2.13. Cell Migration and Invasion Assay

The migration and invasion assay were performed using the modified Boyden chamber method. For the migration assay, polycarbonate filters (pore size 8 µm; Millipore, Carrigtwohill, Tullagreen) free of polyvinylpyrrolidone were coated with 50 µL of fibronectin (10 µg/mL). For the invasion assay, filters were coated with fibronectin (10 µg/mL) and the upper filter was coated with Matrigel (10 µg/50 µL). SaOS-2 cells (1.25 × 10^5^ cells/well) were treated with 0, 1, and 2 μM simvastatin and seeded into the upper chambers. The lower chambers contained medium supplemented with 1% FBS as a chemoattractant. After an 18 h incubation, cells that had migrated or invaded through to the underside of the membrane were fixed in methanol and stained with 1% (*w*/*v*) toluidine blue. The number of cells on the lower membrane surface was subsequently counted under a light microscope.

### 2.14. Gelatin Zymography Assay

Culture supernatants were collected in equal volumes from cells treated with 0–6 μM simvastatin for 24 h in DMEM supplemented with 0.5% serum. The supernatants were separated by electrophoresis on 10% polyacrylamide gels containing 0.1% (*w*/*v*) gelatin under non-reducing conditions. Gels were washed twice with 2.5% Triton X-100 for 30 min at room temperature following electrophoresis to eliminate residual SDS. Gels were then incubated in activation buffer (50 mM Tris–HCl, 200 mM NaCl, 10 mM CaCl_2_, pH 7.4) at 37 °C for 24 h. Following incubation, gels were stained with 0.1% (*w*/*v*) Coomassie Brilliant Blue R and destained using 30% methanol and 10% acetic acid. MMP-2 activity was visualized as clear bands against a blue background and quantified using ImageJ software (v1.410).

### 2.15. Western Blot Analysis

The whole-cell lysate was then separated via SDS-PAGE electrophoresis and transferred to a nitrocellulose membrane via electroblotting. After protein transfer, membranes were blocked and incubated with primary antibodies diluted in one-step blocking solution (Bio-Helix, New Taipei, Taiwan) for 2 h at room temperature. The membranes were subsequently incubated with secondary antibodies (1:20,000 dilution) in the same blocking solution for 1 h. The membranes were then washed five times with PBS containing 5% Tween-20, each for 5 min, and stored in PBS prior to imaging. Protein bands were visualized using enhanced chemiluminescence and captured with the iBright™ CL-1500 imaging system (Thermo Fisher Scientific). Densitometric analysis of band intensity was performed using ImageJ software (version 1.410) to quantify protein expression levels.

### 2.16. Statistical Analysis

All data were from at least three independent experiments. Statistical analysis was performed using Jamovi (Version 2.6). ANOVA and Student’s *t*-test was applied to determine mean differences. Mean differences were considered significant when * *p* < 0.05, ** *p* < 0.01, *** *p* < 0.001.

## 3. Results

### 3.1. Differential Gene Expression Analysis of Osteosarcoma Patients Sensitive and Resistance to Chemotherapy

**Figure 1:**
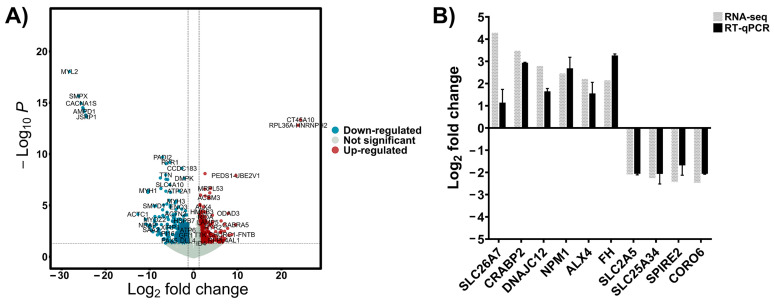
(**A**) Volcano diagram displaying the DEGs between OS samples with good versus poor response to chemotherapy. The diagram highlights 621 upregulated and 423 downregulated genes, with each point representing a gene’s magnitude and significance of differential expression. (**B**) Validation of the RNA sequencing data was performed using RT qPCR to confirm the expression levels of key DEGs, ensuring the accuracy of the bioinformatics analysis.

To investigate the transcriptional differences between patients with a good response to chemotherapy and a poor response, tumor tissues from 9 osteosarcoma patients were subjected to RNA sequencing. Patient’s characteristics are shown in Table S1. DEseq2 was used to identify DEGs between good response and poor response to chemotherapy groups. A total of 15,965 protein-coding genes were detected in Table S2. Among them, 1044 protein-coding genes passed the filtering criteria of |log2FC| > 1.25 and *p*-value < 0.05. Of these, 621 genes were significantly upregulated in the poor response group, while 423 genes were significantly downregulated, as shown in the volcano plot (Figure 1A). Moreover, the top 50 upregulated and downregulated genes are shown in Tables S3 and S4. To validate the RNA-seq results, six upregulated genes and four downregulated genes were randomly selected, and RT-qPCR analysis was performed. As shown in Figure 1B, the expression trends of the selected genes measured by RT-qPCR were consistent with the RNA-seq results.

### 3.2. Functional Enrichment and Protein–Protein Interaction (PPI) Network Analysis of DEGs Associated with Chemotherapeutic Response in OS

**Figure 2:**
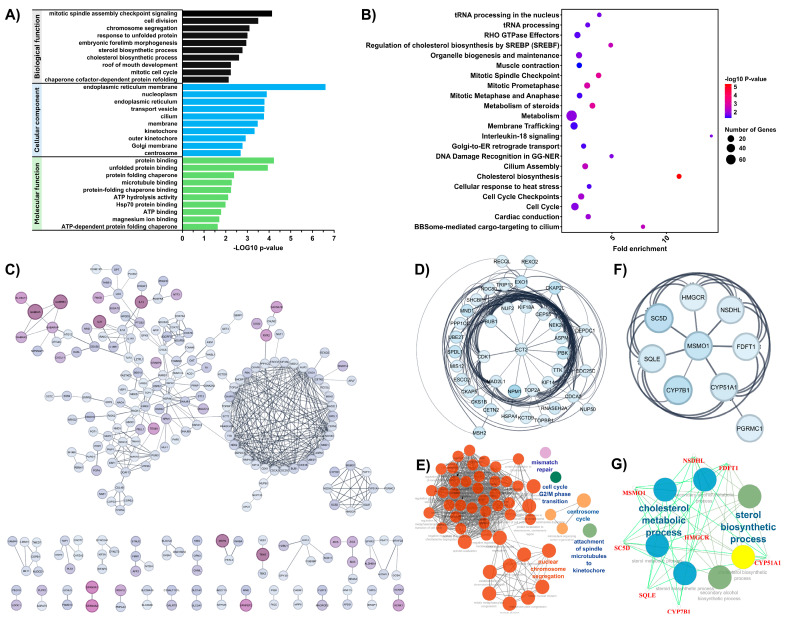
Transcriptomic profiling of drug response-associated pathways in osteosarcoma. (**A**) This panel presented the GO enrichment analysis of the differentially expressed genes (DEGs). It highlighted key biological processes, molecular functions, and cellular compartment associated with drug response in osteosarcoma. (**B**) REACTOME pathway enrichment analysis of DEGs. (**C**) Protein–protein interaction (PPI) network visualized using Cytoscape, where nodes are color-coded by fold-change levels (darker violet indicates higher fold-changes). (**D**) Hub genes obtained from the PPI network were identified using MCODE, showing the most significant module (MCODE score = 11.42). (**E**) Functional enrichment and pathway analysis of the hub genes in panel (**D**) were visualized using ClueGO. (**F**) The second significant module identified by MCODE (MCODE score = 6.0). (**G**) Functional enrichment and pathway analysis of the hub genes in panel (**F**) were visualized using ClueGO.

Gene Ontology (GO) and Reactome pathway enrichment analyses were performed using DAVID to identify significantly enriched biological pathways and functions associated with the chemotherapeutic response in osteosarcoma. Upregulated DEGs were used for the analysis. Significantly enriched GO terms (*p* < 0.05) from each category are presented in Tables S5–S7. The top ten in GO biological process (BP), molecular function (MF), and cellular compartment (CC) are shown in Figure 2A. In the BP category, upregulated DEGs were predominantly enriched in the steroid biosynthetic process, cholesterol biosynthetic process, response to unfolded protein, and several cell cycle–related processes, including mitotic spindle assembly checkpoint signaling, cell division, chromosome segregation, and the mitotic cell cycle. In the CC category, these genes were mainly associated with the endoplasmic reticulum, nucleoplasm, and kinetochore. In the MF category, the most significantly enriched terms included protein binding, unfolded protein binding, microtubule binding, and ATP hydrolysis activity. Figure 2B shows that Reactome pathway analysis (*p* < 0.05) revealed significant enrichment in cholesterol biosynthesis and mitotic cell cycle-related pathways, including resolution of sister chromatid cohesion, mitotic spindle checkpoint, and mitotic prometaphase. Additionally, pathways involving RHO GTPase effectors were also significantly enriched.

To determine the interactions among differentially expressed proteins associated with the chemotherapeutic response in osteosarcoma, a PPI network was constructed by importing upregulated DEGs into the STRING database with a minimum confidence score of 0.7 and visualized using Cytoscape. The network diagram contains 516 nodes and 490 edges (Figure 2C). MCODE was used to identify and visualize clusters of genes within the PPI network. Subsequently, the ClueGO plugin in Cytoscape was employed to interpret non-redundant biological terms associated with each identified cluster. Two clusters with MCODE scores greater than 5 were identified. The top-scoring gene cluster (MCODE score = 11.42) was significantly associated with nuclear chromosome segregation, mismatch repair, centrosome cycle, G2/M phase transition of the cell cycle, and spindle microtubule attachment to kinetochores (Figure 2D,E). The second cluster (MCODE score = 6.0) was significantly enriched in sterol biosynthetic processes and cholesterol metabolism (Figure 2F,G). These findings suggest that cholesterol biosynthesis and mitotic cell cycle processes may contribute to the chemotherapeutic response of OS to the MAP regimen.

### 3.3. The Role of the Cholesterol Biosynthesis Pathway in the Chemotherapeutic Drug Response of Osteosarcoma Cells

**Figure 3:**
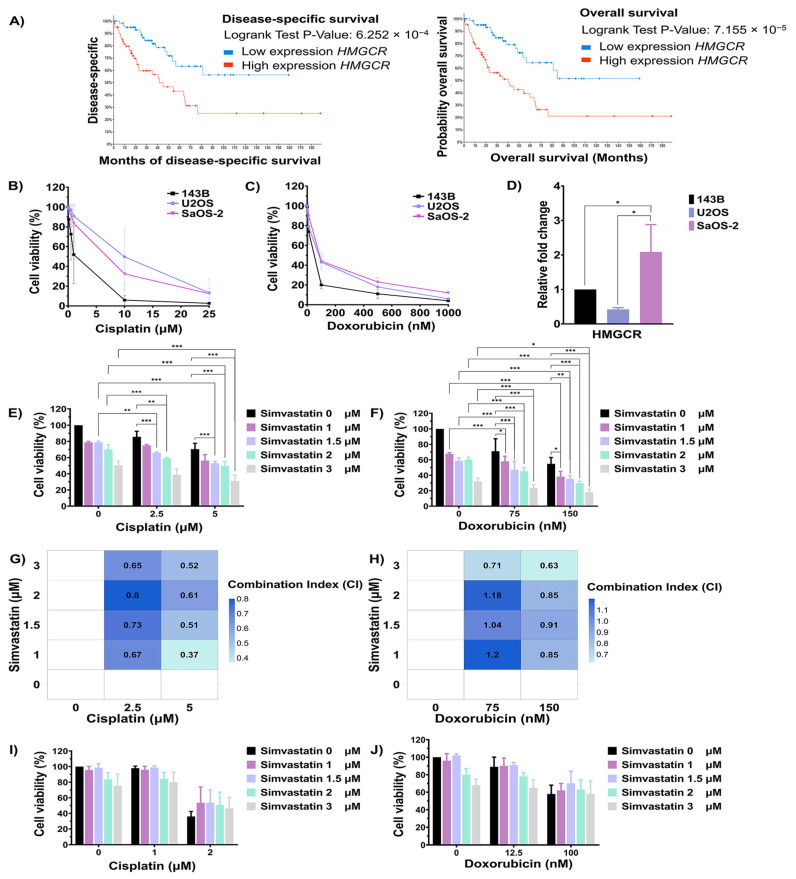
Evaluation of the cholesterol biosynthesis pathway in modulating chemotherapeutic response in osteosarcoma cells. (**A**) Kaplan–Meier curves showing overall survival and disease-specific survival of sarcoma patients stratified by *HMGCR* expression, analyzed using cBioPortal. (**B**) Cell viability of OS cell lines after cisplatin treatment for 72 h was assessed using the MTT assay. (**C**) Cell viability of OS cell lines after doxorubicin treatment for 72 h was assessed using the MTT assay. (**D**) Expression of the *HMGCR* gene in OS cell lines was evaluated using RT-qPCR. Effect of simvastatin in combination with cisplatin (**E**) or doxorubicin (**F**) on SaOS-2 cell viability, as determined by MTT assay. (**G**) The Heatmap with CI values of a combination between cisplatin and simvastatin in SaOS-2 cells. (**H**) The Heatmap with CI values of a combination between doxorubicin and simvastatin in SaOS-2 cells. A CI value of < 0.80 indicates synergism, 0.80–1.20 indicates an additive effect, and > 1.20 indicates antagonism. Effect of simvastatin in combination with cisplatin (**I**) or doxorubicin (**J**) on 143B cell viability. All experiments were repeated at least three times. * *p* < 0.05, ** *p* < 0.01, and *** *p* < 0.001 compared to the control group.

Pathway enrichment analyses highlighted cholesterol biosynthesis as the most significantly enriched pathway associated with chemotherapeutic response in OS. To further investigate the relationship between key regulatory genes involved in cholesterol biosynthesis and clinical outcomes, overall survival, and disease-specific data from sarcoma patients in the TCGA, the PanCancer Atlas database was analyzed using cBioPortal. As shown in Figure 3A, the upregulation of *HMGCR* was significantly associated with poor overall survival and disease-specific survival in sarcoma tissue. To elucidate its functional relevance, in vitro studies were conducted using OS cell lines to investigate the role of cholesterol biosynthesis in modulating cellular responses to chemotherapeutic agents. The sensitivity of 143B, U2OS, and SaOS-2 cells to doxorubicin and cisplatin was assessed using the MTT assay. Cells were treated with varying concentrations of cisplatin and doxorubicin for 72 h. The inhibitory concentration at 50% (IC_50_) values for cisplatin in 143B, U2OS, and SaOS-2 cells were 1.37 μM, 9.92 μM, and 6.90 μM, respectively (Figure 3B). In contrast, the IC_50_ values for doxorubicin in 143B, U2OS, and SaOS-2 cells were 50.42 nM, 92.52 nM, and 98.98 nM, respectively (Figure 3C). Furthermore, the expression level of the *HMGCR* gene in OS cell lines was determined using qRT-PCR. As shown in Figure 3D, the expression level of *HMGCR* was significantly highest in SaOS-2 cells compared to 143B and U2OS cells. Based on both *HMGCR* expression levels and the IC_50_ values for chemotherapeutic agents, 143B cells were selected as a model for good drug response, while SaOS-2 cells were chosen to represent poor drug response for investigating the role of the cholesterol biosynthesis pathway in modulating drug sensitivity.

To evaluate the role of cholesterol biosynthesis in regulating the chemotherapeutic response, the cholesterol biosynthesis inhibitor simvastatin was used. 143B and SaOS-2 cells were treated with a combination of simvastatin and either cisplatin or doxorubicin. As shown in Figure 3E, treatment of SaOS-2 cells with 2.5 and 5 µM cisplatin for 72 h reduced cell viability to 85.62% and 70.41%, respectively. In contrast, combination treatment with 5 µM cisplatin and simvastatin at,1.5, 2, and 3 µM significantly reduced viability to 53.03%, 49.67%, and 31.08%, respectively, compared to treatment with simvastatin or cisplatin alone. Similarly, when SaOS-2 cells were treated with 2.5 mM of cisplatin, the addition of simvastatin at 1 mM and 1.5 mM significantly decreased cell viability to 75.03% and 65.84%, respectively. In parallel, treatment of SaOS-2 cells with 150 nM doxorubicin reduced cell viability to 54.73%, while co-treatment with simvastatin at 1.5, 2, and 3 µM further decreased viability to 35.26%, 29.62%, and 17.90%, respectively, compared to either agent alone (Figure 3F). To evaluate the nature of the drug interaction, combination index (CI) analysis was performed. A CI value of <0.80 indicates synergism, 0.80–1.20 indicates an additive effect, and >1.20 indicates antagonism. As shown in Figure 3G and Figure 3H, combination treatment of simvastatin (1–3 µM) with 2.5 or 5 µM cisplatin demonstrated a synergistic effect, as indicated by CI values below 0.80. Similarly, co-treatment with simvastatin at 2 µM and doxorubicin at either 75 or 150 nM also yielded CI values below 0.80, indicating synergism. In contrast, combination treatments involving simvastatin at 1, 1.5, and 2 µM with doxorubicin at 75 or 150 nM showed CI values ranging from 0.80 to 1.20, consistent with an additive effect. On the other hand, the treatment of 143B cells with 2 µM cisplatin reduced cell viability to 62.37%, whereas combination treatment with 2 µM cisplatin and simvastatin at 2 and 3 µM increased cell viability to 86.87% and 76.73%, respectively. In 143B cells, treatment with 100 nM doxorubicin lowered viability to 58.29% and co-treatment with simvastatin did not significantly alter viability compared to doxorubicin alone (Figure 3I,J). In addition, combination treatment of cisplatin and simvastatin did not significantly induced U2OS cell death when compared to either treatment alone (Figure S1). These results indicate that the inhibition of cholesterol biosynthesis by simvastatin enhances the sensitivity of SaOS-2 cells, but not 143B cells, to cisplatin and doxorubicin.

### 3.4. Simvastatin Enhance Cisplatin and Doxorubicin-Induced SaOS-2 Cells Apoptosis

**Figure 4:**
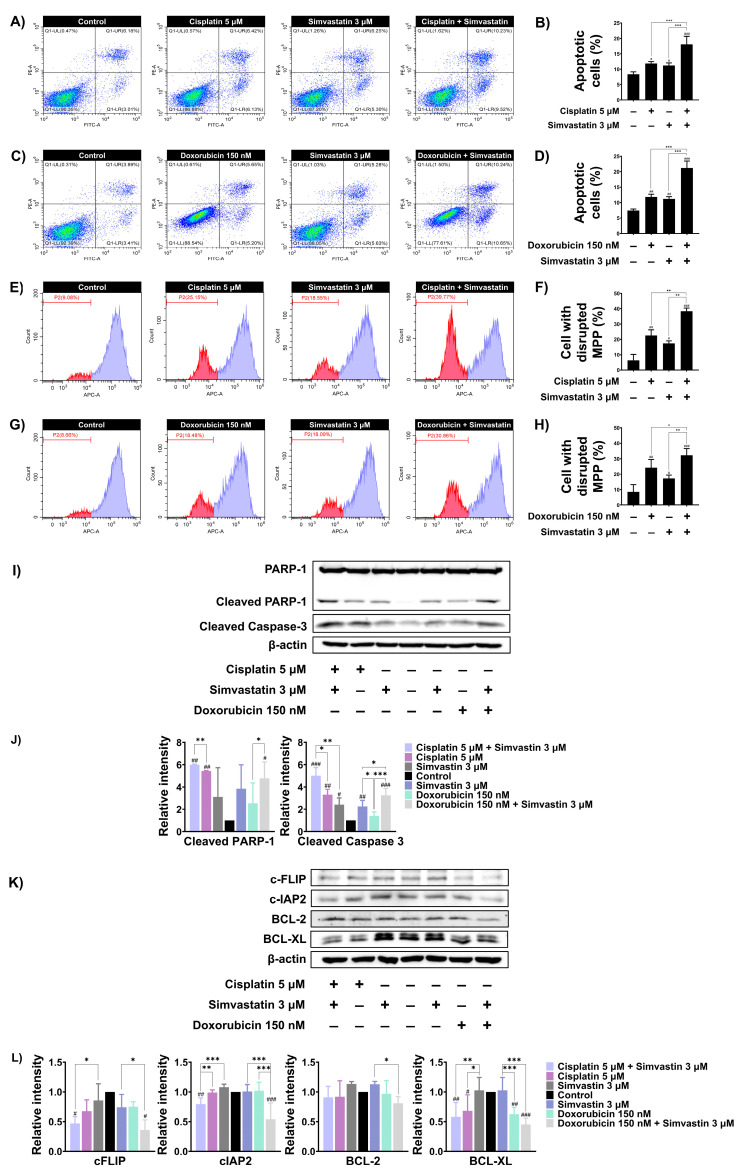
The effect of inhibiting cholesterol biosynthesis on apoptotic alterations in SaOS-2 cells. (**A**) SaOS-2 cells were treated with 5 μM cisplatin with or without 3 μM simvastatin for 48 h. Apoptosis was assessed using Annexin V-FITC/PI staining followed by flow cytometry. (**B**) Data are presented as a bar graph. (**C**) SaOS-2 cells were treated with 150 nM doxorubicin with or without 3 μM simvastatin for 48 h and apoptosis was analyzed. (**D**) Data are presented as a bar graph. (**E**) MMP changes after combination treatment with 5 μM cisplatin and 3 μM simvastatin were assessed using MitoView™ staining and flow cytometry was used for analysis. (**F**) Data are presented as a bar graph. (**G**) MMP changes following combination treatment of 150 nM doxorubicin and 3 μM simvastatin for 48 h were analyzed similarly. (**H**) Data are presented as a bar graph. (**I**) Western blot analysis of cleaved PARP-1 and caspase-3 levels in SaOS-2 cells treated with simvastatin in combination with either cisplatin or doxorubicin for 36 h. (**J**) Data are presented as a bar graph. (**K**) Expression of anti-apoptotic proteins (BCL-2, BCL-XL, c-FLIP, and c-IAP2) was evaluated under the same conditions. (**L**) Data are presented as a bar graph. * *p* < 0.05, ** *p* < 0.01, and *** *p* < 0.001 (compared within experimental groups). # *p* < 0.05, ## *p* < 0.01, and ### *p* < 0.001 (compared to control group). All data are representative of three independent experiments as mean ± SD.

We next investigated whether simvastatin potentiates cisplatin and/or doxorubicin induced cell death by enhancing apoptosis. Based on the CI values obtained from previous experiments, we selected concentrations of simvastatin and chemotherapeutic agents that exhibited synergistic interactions to further evaluate the molecular mechanisms underlying cell death. SaOS-2 cells were treated with chemotherapeutic agents, simvastatin alone, or a combination of both, and apoptosis was assessed using Annexin V/PI staining. As shown in Figure 4A and Figure 4B, combination treatment with 5 mM cisplatin and 3 µM simvastatin significantly increased apoptosis to 18.06% compared to treatment with cisplatin or simvastatin alone. Similarly, combination treatment with 150 nM doxorubicin and 3 µM simvastatin significantly increased the apoptotic population to 21.21% compared to doxorubicin or simvastatin treatment alone (Figure 4C,D). It is well established that cisplatin and doxorubicin induce apoptosis through the intrinsic pathway by disrupting mitochondrial membrane potential (MMP). To assess changes in MMP, SaOS-2 cells were stained with MitoView™ 633, a membrane-permeable dye that fluoresces brightly upon accumulation within mitochondria in a membrane potential-dependent manner. Alterations in MMP were subsequently analyzed by flow cytometry. As shown in (Figure 4E,F), treatment with 5 μM of cisplatin or 3 μM of simvastatin alone caused a significant disruption of MMP in SaOS-2 cells to 22.63 and 17.41%, respectively. However, combination treatment with cisplatin and simvastatin further increased MMP loss to 38.38%. Similarly, treatment with 150 nM doxorubicin alone reduced MMP to 24.25%, whereas co-treatment with doxorubicin and simvastatin significantly increased MMP disruption to 32.25% compared to either treatment alone (Figure 4G,H). Furthermore, apoptosis-related proteins were assayed by Western blotting. The level of cleaved PARP-1 and caspase-3 levels induced by doxorubicin or cisplatin alone were significantly enhanced by combination treatment with simvastatin (Figure 4I,J). Anti-apoptotic proteins such as c-FLIP, c-IAP2, BCL-2, and BCL-XL are known to regulate cisplatin- and doxorubicin-induced apoptosis. Combination treatment with simvastatin and either cisplatin or doxorubicin markedly reduced the expression of c-FLIP and c-IAP2 compared to simvastatin alone. Notably, co-treatment with simvastatin and either cisplatin or doxorubicin also decreased BCL-XL expression when compared to simvastatin alone. Likewise, combination treatment of simvastatin and doxorubicin significantly reduced BCL-2 expression when compared to simvastatin alone, whereas combination with cisplatin did not significantly alter its expression (Figure 4K,L). These findings suggest that the cholesterol biosynthesis pathway may, at least in part, be involved in modulating the apoptotic sensitivity of SaOS-2 cells to cisplatin and doxorubicin through the downregulation of anti-apoptotic proteins.

### 3.5. Simvastatin Reduced SaOS-2 Invasion and Migration

**Figure 5:**
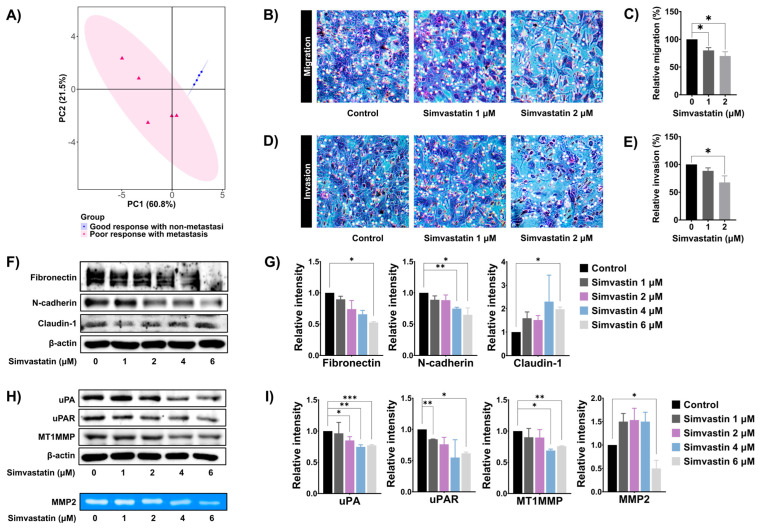
Role of cholesterol biosynthesis pathway in SaOS-2 cells migration and invasion. (**A**) PCA score plot showing the association between the expression of cholesterol and steroid biosynthesis-related genes and metastasis status in OS tissues. (**B**) Boyden chamber assay was performed to detect the migration of SaOS-2 after treatment with simvastatin for 18 h (10× magnifications). (**C**) Quantification of migrated cells represented as a bar graph. (**D**) The effects of simvastatin on SaOS-2 cell invasion were evaluated by using Boyden camber assay after treatment with simvastatin for 18 h (10× magnifications). (**E**) Quantification of invaded cells are represented as a bar graph. (**F**) Western blot analysis of epithelial–mesenchymal transition (EMT)-related protein expression in SaOS-2 cells following 24 h simvastatin treatment. (**G**) Densitometric quantification of EMT protein bands using ImageJ, presented as histograms. (**H**) Western blot analysis of extracellular matrix (ECM)-degrading enzyme expression in SaOS-2 cells after 24 h of simvastatin exposure. (**I**) Quantification of ECM-related protein band intensity using ImageJ, presented as a histogram. Results were mean ± S.D. of three independent experiments. * *p* < 0.05, ** *p* < 0.01, *** *p* < 0.001 compared to control. All data are representative of three independent experiments as mean ± SD.

Next, principal component analysis (PCA) was performed to investigate the association between the expression levels of cholesterol and steroid biosynthesis-related genes and metastasis in our tissue samples. The PCA revealed a distinct separation between metastatic and non-metastatic OS tissue samples based on the significant expression profiles of cholesterol and steroid biosynthesis-related genes (Figure 5A). To investigate the functional role of the cholesterol biosynthesis pathway in metastatic properties of OS, SaOS-2 cells were treated with simvastatin, and cell migration and invasion were assessed using the Boyden chamber assay. As shown in Figure 5B–E, simvastatin at 2 mM significantly decreased SaOS-2 cell migration and invasion. Epithelial–mesenchymal transition (EMT) is a key mechanism that facilitates tumor cell motility and invasion. To evaluate whether simvastatin affects EMT, the expression levels of EMT-related proteins—including fibronectin, N-cadherin, and claudin-1—were assessed by Western blot analysis. Treatment of SaOS-2 cells with 6 mM simvastatin for 18 h significantly decreased the levels of mesenchymal markers fibronectin and N-cadherin in a dose-dependent manner. In contrast, simvastatin significantly upregulated the epithelial marker claudin-1 (Figure 5F and Figure 5G). In addition, we examined the effects of simvastatin on the expression of extracellular matrix (ECM)-degrading proteins in SaOS-2 cells. As shown in Figure 5H and Figure 5I, simvastatin at 6 mM significantly reduced the expression levels of uPA, uPAR, MT1-MMP, and MMP-9 in a dose-dependent manner. Collectively, these results indicate that the cholesterol biosynthesis pathway may regulate the metastatic potential of OS by modulating EMT and ECM remodeling.

### 3.6. Simvastatin Modulates Ras Prenylation and Downstream Signaling in SaOS-2 Cells

**Figure 6:**
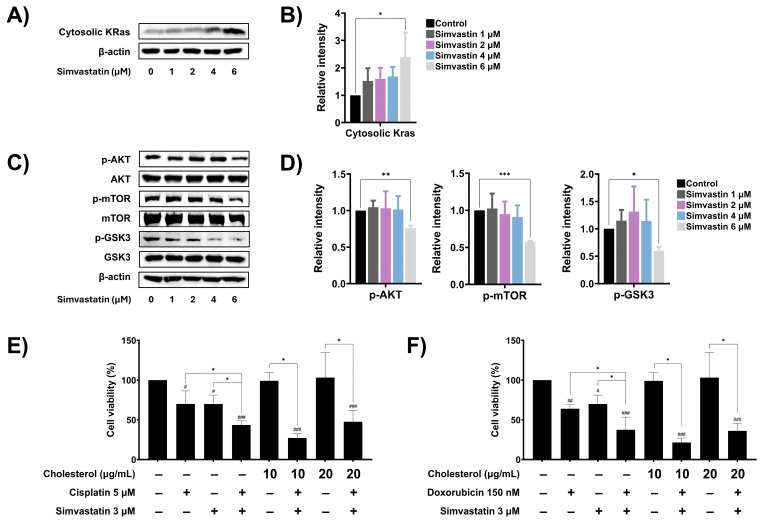
The role of cholesterol biosynthesis pathway on the modulation of oncogenic signaling. (**A**) Effect of simvastatin on cytosolic Ras, after being treated with simvastatin for 24 h. (**B**) Band density was quantified using ImageJ and presented as a histogram. (**C**) Effect of simvastatin on expression levels of phosphorylated and non-phosphorylated forms of cellular Akt, mTOR, and GSK3 signaling proteins, after being treated with simvastatin for 24 h. (**D**) Band density was quantified using ImageJ and presented as a histogram. The Western blot results are representative of three independent experiments. Effect of cholesterol levels on SaOS-2 cell viability during treatment with cisplatin (**E**) or doxorubicin (**F**). Exogenous cholesterol was added to cells co-treated with cisplatin and simvastatin or doxorubicin and simvastatin. Cell viability was assessed using MTT assay. All experiments were performed in triplicate. * *p* < 0.05, ** *p* < 0.01, and *** *p* < 0.001 (compared within experimental groups). # *p* < 0.05, ## *p* < 0.01, and ### *p* < 0.001 (compared to control group).

Cholesterol biosynthesis has been implicated in cancer cell metastasis and resistance to chemotherapeutic drugs, in part through the prenylation of oncogenic signaling molecules such as Ras. Once prenylated, Ras translocates to the plasma membrane, where it becomes activated and initiates downstream signaling cascades, including the Akt/mTOR and Akt/GSK3 pathways, which promote the expression of anti-apoptotic, survival, and metastasis-related proteins. To determine whether simvastatin regulates Ras function by inhibiting prenylation, the cytoplasmic levels of Ras were assessed by Western blot analysis. Treatment of SaOS-2 cells with increasing concentrations of simvastatin significantly elevated the cytoplasmic levels of Ras in a dose-dependent manner (Figure 6A,B), suggesting inhibition of Ras prenylation and membrane localization. Next, the effect of simvastatin on downstream Ras signaling pathways including AKT/mTOR and AKT/GSK3 was investigated. As shown in Figure 6C and Figure 6D, treatment with 6 µM simvastatin significantly reduced the phosphorylation of Akt, mTOR, and GSK-3, indicating suppression of the AKT/mTOR and AKT/GSK3 signaling cascades. To further evaluate whether cholesterol levels contribute to the drug response in SaOS-2 cells, exogenous cholesterol was added to the combination treatment with simvastatin and chemotherapeutic agents, and cell viability was assessed using the MTT assay. As shown in Figure 6E, treatment with cholesterol alone had no effect on cell viability. Co-treatment with exogenous cholesterol (20 µg/mL), cisplatin, and simvastatin reduced cell viability to 47.47%, which was not significantly different from the combination treatment of cisplatin and simvastatin (43.43%). Similarly, co-treatment with exogenous cholesterol, doxorubicin, and simvastatin did not significantly alter cell viability compared to the combination treatment of doxorubicin and simvastatin (Figure 6F). These findings suggest that simvastatin exerts its chemosensitizing effects on SaOS-2 cells by inhibiting Ras prenylation and suppressing key oncogenic signaling pathways, rather than by altering intracellular cholesterol levels.

## 4. Discussion

Neoadjuvant chemotherapy with the MAP regimen is the standard treatment for high-grade osteosarcoma, offering the benefit of reducing primary tumor size and eliminating micrometastases before surgery [2]. However, chemoresistance remains a major obstacle, contributing to poor patient outcomes. The percentage of tumor necrosis is the most used as a marker of chemotherapy response, but it requires post-surgical evaluation and offers limited utility for early treatment decisions. Therefore, identifying molecular pathways associated with chemotherapy response is essential for improving prognosis and guiding more effective, individualized treatment strategies.

In the present study, RNA-seq was performed to compare transcriptional profiles of pre-chemotherapy OS patient samples exhibiting differential responses to chemotherapy, with the aim of identifying the pathways associated with therapeutic outcomes. The upregulated DEGs were subsequently analyzed using GO and Reactome pathway enrichment to identify significantly enriched biological pathways and functions relevant to the chemotherapeutic response. GO analysis indicated that the upregulated DEGs were significantly enriched in biological processes related to cell cycle regulation, steroid biosynthesis, and response to unfolded protein. Similarly, Reactome pathway analysis identified cholesterol biosynthesis as the most significantly enriched pathway, followed by the mitotic spindle checkpoint pathway. Notably, overexpression of genes involved in the mitotic spindle checkpoint, such as *NUF2*, *MAD2L1*, and *BUB1*, has been linked to chemotherapeutic responses in various cancer types [11,12,13,14,15,16,17,18,19,20]. In addition, activation of the cholesterol biosynthesis pathway has been shown to promote cancer progression in multiple malignancies, including breast, ovarian, and liver cancers [21,22,23,24]. Conversely, the inhibition of cholesterol biosynthesis has been reported to sensitize cancer cells to chemotherapeutic agents such as cisplatin and doxorubicin [25,26,27,28,29]. These findings were supported by the PPI network generated using STRING, which revealed two major functional clusters identified by MCODE. The top-scoring cluster was enriched in genes associated with mitotic regulation and chromosomal dynamics, while the second cluster showed strong enrichment in sterol biosynthetic processes and cholesterol metabolism. These results suggest that both mitotic progression and dysregulation of cholesterol biosynthesis may play pivotal roles in mediating resistance to MAP chemotherapy in osteosarcoma, highlighting potential targets for therapeutic intervention.

Numerous studies have indicated the cholesterol biosynthesis pathway is upregulated in various types of tumors [30,31,32]. Notably, overexpression of *HMGCR*, the rate-limiting enzyme in endogenous cholesterol synthesis, has been reported in several cancers, including gastric, bladder, and liver tumors [33,34,35]. Interestingly, the inhibition of *HMGCR* using statins has demonstrated beneficial effects when used in combination with chemotherapy [36,37,38,39,40]. In agreement with these observations, we demonstrated that high expression of *HMGCR* is associated with poor overall survival and disease-specific in sarcoma tissue. In our functional validation, SaOS-2 cells, characterized by high *HMGCR* expression and drug resistance, demonstrated increased sensitivity to cisplatin and doxorubicin upon co-treatment with simvastatin, a cholesterol biosynthesis inhibitor. This synergistic effect was confirmed through combination index analysis. Conversely, 143B cells with lower *HMGCR* expression did not exhibit enhanced chemosensitivity upon simvastatin co-treatment, suggesting a context-dependent effect of cholesterol biosynthesis inhibition. Interestingly, U2OS cells exhibited a similar sensitivity profile to chemotherapeutic drugs as SaOS-2 cells, despite expressing lower levels of *HMGCR*. However, simvastatin did not markedly enhance cisplatin sensitivity in U2OS cells. Moreover, the IC_50_ of simvastatin in SaOS-2 cells was approximately 3 μM, whereas in U2OS cells, it exceeded 10 μM, indicating lower susceptibility. This observation aligns with previous reports suggesting that the mevalonate pathway plays a critical role in the survival and metastasis of *TP53* mutant cancers [41,42]. Since SaOS-2 cells are p53-null, while U2OS cells are wild-type p53, these findings suggest that cholesterol biosynthesis may contribute more significantly to chemotherapeutic drug sensitivity in p53-deficient OS cells, whereas it may play a lesser role in wild-type p53 U2OS cells. Collectively, these results further support the involvement of the cholesterol biosynthesis pathway in modulating chemotherapeutic drug response in osteosarcoma.

Previous studies have shown that statins can induce cancer cell death by promoting apoptosis [43,44]. Therefore, we investigated whether simvastatin enhances the sensitivity of SaOS-2 cells to chemotherapeutic agents through an apoptotic mechanism. Co-treatment with simvastatin and either cisplatin or doxorubicin significantly increased the proportion of apoptotic cells. Consistent with these findings, Western blot analysis revealed that simvastatin enhanced cisplatin- and doxorubicin-induced apoptosis by increasing the levels of cleaved PARP and cleaved caspase-3, confirming the activation of apoptotic cell death. Mitochondrial dysfunction is a key trigger of the intrinsic apoptotic pathway. Anticancer drugs can disrupt MMP by increasing outer membrane permeability, leading to impaired ATP production, elevated ROS, cytochrome c release, and apoptosis [45]. This process is tightly regulated by Bcl-2 family proteins [46]. Anti-apoptotic members such as Bcl-2 and Bcl-xL maintain mitochondrial integrity, while c-FLIP and c-IAP2 inhibit caspase activation [47]. Overexpression of these proteins is commonly observed in various cancers and contributes to resistance to apoptosis. Accordingly, their downregulation can sensitize tumor cells to chemotherapy. Consistent with the above-mentioned studies, combination treatment with simvastatin and chemotherapeutic agents induced significant mitochondrial dysfunction, as evidenced by increased MMP loss. Furthermore, the expression of key anti-apoptotic proteins, including c-FLIP, c-IAP2, and BCL-XL, was markedly reduced following combination treatment. Alignment with previous studies indicate that statins have been shown to decrease the protein levels of anti-apoptotic proteins such as Bcl-2 and Bcl-xL. This suggests that simvastatin enhances mitochondrial-mediated apoptosis by downregulating survival pathways.

Metastasis is a leading cause of cancer-related mortality in high-grade OS. A key event in this process is the disruption of cellular integrity and interaction with the extracellular matrix (ECM), which facilitates tumor cell metastasis. Epithelial–mesenchymal transition (EMT) plays a crucial role in cancer invasion and metastasis by enabling epithelial cells to lose polarity and adhesion to gain migratory and invasive mesenchymal characteristics. This transition involves decreased expression of epithelial markers like E-cadherin and increased levels of mesenchymal markers such as N-cadherin and fibronectin [48]. In parallel, disruption of the interaction between cancer cells and ECM integrity facilitates metastasis through ECM-degrading enzymes such as MMPs and uPA [49]. Emerging evidence has implicated cholesterol biosynthesis in promoting tumor metastasis [50]. In our study, PCA revealed distinct expression profiles of cholesterol and steroid biosynthesis genes between metastatic and non-metastatic OS samples, suggesting a strong association between this pathway and OS metastasis. Functional assays further demonstrated that simvastatin significantly reduced migration and invasion of SaOS-2 cells. Mechanistically, simvastatin downregulated mesenchymal markers fibronectin, while N-cadherin upregulated the epithelial marker claudin-1 and suppressed the expression of ECM-degrading enzymes, including uPA, uPAR, MT1-MMP, and MMP-9. These results aligned with previous findings showing that statins reduce tumor invasiveness in various cancer types by targeting EMT and ECM remodeling [51,52]. Collectively, these findings suggest that targeting cholesterol biosynthesis may offer a promising strategy to suppress OS metastasis by concurrently inhibiting EMT and ECM degradation.

HMGCR, the rate-limiting enzyme in the mevalonate pathway, not only regulates cholesterol biosynthesis but also produces essential isoprenoid intermediates such as farnesyl pyrophosphate (FPP) and geranylgeranyl pyrophosphate (GGPP). These isoprenoids are critical for protein prenylation, a post-translational modification required for the membrane localization and function of small GTPases, including Ras and Rho family proteins [53]. Prenylation enables Ras proteins to translocate from cytoplasm to the plasma membrane, where they activate downstream pathways such as Akt/mTOR and Akt/GSK3, which promote cell proliferation, survival, and metastasis [54]. Consistent with this mechanism, our findings demonstrated that simvastatin treatment in SaOS-2 cells significantly increased cytoplasmic Kras levels, indicating impaired prenylation and reduced membrane localization. These results align with previous studies, reporting that statins reduce Ras membrane association in leukemic and breast cancer cells, thereby promoting apoptosis. Moreover, we investigated the effect of simvastatin on Ras downstream signaling pathways, including Akt/mTOR and Akt/GSK3, which regulate the expression of genes involved in cell proliferation, survival, and metastasis in SaOS-2 cells. In the present study, simvastatin markedly reduced the activity of Akt, mTOR, and GSK-3 by decreasing the phosphorylation level. This is consistent with studies indicating that statins inhibited Ras prenylation and Ras signaling downstream effectors, including Akt, GSK3, and mTOR, thereby inducing apoptosis and metastasis [55]. Importantly, our rescue experiments with exogenous cholesterol failed to reverse the cytotoxic effects of simvastatin when combined with cisplatin or doxorubicin, suggesting that the chemosensitizing effects of statins are not primarily mediated by intracellular cholesterol depletion. Rather, our data support the model in which simvastatin exerts its anti-tumor activity by disrupting the mevalonate-derived prenylation of signaling proteins, particularly Ras, thereby inhibiting oncogenic signaling and enhancing apoptosis. This mechanistic insight aligns with reports that statin-induced apoptosis can be rescued by GGPP or FPP, but not by sterols [56]. Our findings provide further evidence that the cholesterol biosynthesis pathway, particularly the mevalonate–Ras prenylation axis, plays a critical role in regulating chemotherapeutic response and metastasis in osteosarcoma cells.

## 5. Conclusions

This study demonstrates that dysregulation of the cholesterol biosynthesis pathway, particularly the mevalonate–Ras prenylation, contributes to chemoresistance and metastasis in OS. Transcriptomic analysis revealed enrichment of cholesterol biosynthesis and mitotic pathways in poor responders, and high *HMGCR* expression, analyzed using TCGA data, was associated with poor prognosis of sarcoma. Functional validation using simvastatin in SaOS-2 cells confirmed its ability to enhance the efficacy of cisplatin and doxorubicin by promoting mitochondrial-mediated apoptosis and suppressing anti-apoptotic proteins. Simvastatin also inhibited cell migration and invasion by reducing EMT and ECM-degrading enzymes. Mechanistically, simvastatin inhibited Ras prenylation and downstream signaling, independent of cholesterol depletion. Our next step is to investigate the correlation between cholesterol biosynthesis gene expression and drug response in high-grade OS tissue. These findings support cholesterol biosynthesis as a critical pathway in OS drug resistance and metastasis.

## Data Availability

The original contributions presented in this study are included in the article and Supplementary Materials. Further inquiries can be directed at the corresponding author, due to (specify the reason for the restriction, e.g., privacy, legal or ethical reasons).

## Supplementary Materials

The following supporting information can be downloaded at: https://www.mdpi.com/article/10.3390/cells14130993/s1. Table S1: Metadata of osteosarcoma tissue sample; Table S2: Differential gene expression of OS sample; Table S3: Top 50 upregulated genes; Table S4: Top 50 downregulated genes; Table S5: GO biological process; Table S6: GO cellular compartment; Table S7: GO molecular function; Figure S1: Chemosensitizing effect of simvastatin on U2OS cell line.

## Abbreviations

The following abbreviations are used in this manuscript:

HGOS	High-grade osteosarcoma
OS	Osteosarcoma
HMGCR	HMG-CoA reductase
DEGs	Differentially expressed genes
GO	Gene Ontology
MMP	Mitochondrial membrane potential
EMT	Epithelial–mesenchymal transition
mTOR	Mechanistic target of rapamycin
GSK3	Glycogen synthase kinase-3
uPA	Urokinase plasminogen activator
