# Supplementary figures and images for: The Cholesterol Biosynthesis Pathway Plays an Important Role in Chemotherapeutic Drug Response and Metastasis in High-Grade Osteosarcoma
Source: Cells. 2025 Jun 29;14(13):993. doi: 10.3390/cells14130993 (PMC12249065; doi:10.3390/cells14130993)

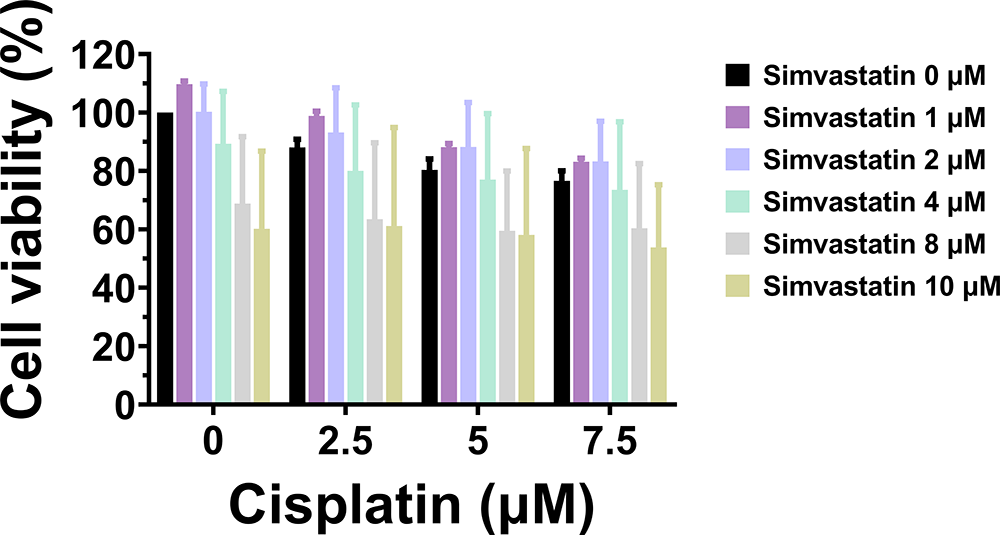

Supplement: Supplementary file 1 [file cells-14-00993-s001.zip › FigureS1-(MTT-Cisplatin-Sim-U2OS) edited.tif]
